# The Immediate Cardiovascular and Mitochondrial Response in Ischemic Cardiogenic Shock

**DOI:** 10.1007/s12265-025-10647-6

**Published:** 2025-06-24

**Authors:** Oskar Kjærgaard Hørsdal, Peter Hartmund Frederiksen, Ole Kristian Lerche Helgestad, Hanne Berg Ravn, Jacob Eifer Møller, Henrik Wiggers, Roni Ranghøj Nielsen, Nigopan Gopalasingam, Kristoffer Berg-Hansen

**Affiliations:** 1 Department of Clinical Medicine, Aarhus University, Aarhus, Denmark; 2 Department of Cardiology, Aarhus University Hospital, Palle Juul Jensens Boulevard 99, 8200 Aarhus N, Denmark; 3 Department of Cardiology, Gødstrup Hospital, Gødstrup, Denmark; 4 Department of Cardiology, Odense University Hospital, Odense, Denmark; 5 Department of Clinical Pharmacology, Aarhus University Hospital, Aarhus, Denmark; 6 Department of Anesthesiology and Intensive Care, Odense University Hospital, Odense, Denmark; 7 Department of Clinical Research, University of Southern Denmark, Odense, Denmark; 8 Heart Center, Copenhagen University Hospital, Rigshospitalet, , Copenhagen, Denmark

**Keywords:** Myocardial ischemia, Cardiogenic shock, Hemodynamics, Mitochondrial function, Pressure–volume

## Abstract

The acute pathophysiological changes after myocardial ischemia complicated by cardiogenic shock (CS) remain poorly defined, especially regarding compensatory mechanisms and myocardial mitochondrial function. We investigated immediate cardiovascular and mitochondrial effects in a porcine model of ischemic CS. CS was induced in 32 Danish Landrace pigs (60 kg) via repeated microembolization of the left coronary artery until a 30% reduction in cardiac output (CO) or mixed venous saturation. Monitoring included pulmonary artery and left ventricular pressure–volume catheters, with analysis of endomyocardial biopsies and arterial, mixed venous, and coronary sinus blood samples. CO deteriorated promptly due to decreased stroke volume. Contractility declined, and afterload increased, causing rapid ventriculo-arterial decoupling. Forward flow parameters were compromised prior to pressure-parameters. Diastolic function was impaired and mitochondrial damage was observed. CS rapidly impairs LV hemodynamic and mitochondrial function, highlighting the importance of monitoring forward flow and targeting mitochondrial function in treatment.

**Graphical Abstract:**

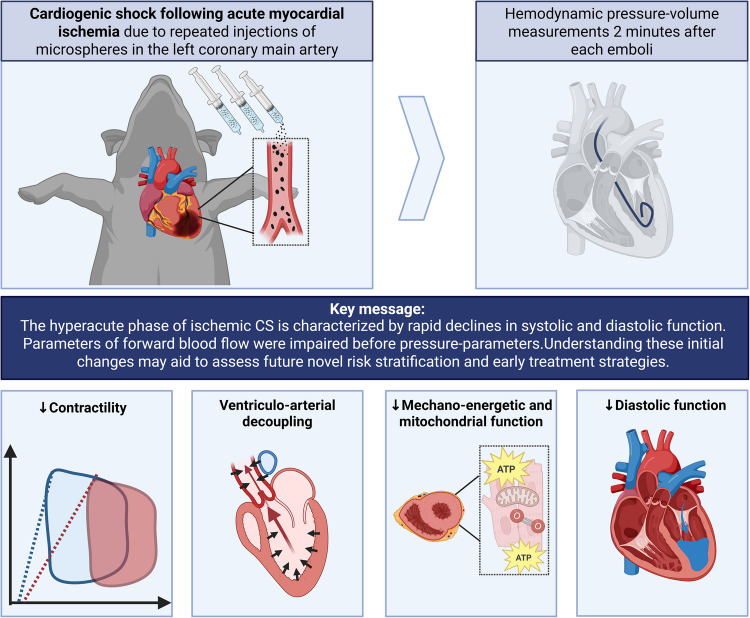

## Background

Acute myocardial infarction is a common cardiac emergency associated with substantial morbidity and mortality [1] and is caused by coronary arterial obstruction and subsequent myocardial ischemia. Despite major improvements in treatment [2] the condition remains a leading cause of cardiogenic shock (CS) and cardiac arrest [3, 4]. Therefore, rapid clinical evaluation of these patients is imperative to identify patients at risk of hemodynamic deterioration. Furthermore, subsequent adverse remodeling due to myocardial necrosis, fibrosis, and inflammation, resulting in structural alterations, is a major cause of heart failure [5, 6]. As such, understanding the early pathophysiological trajectory during acute myocardial ischemia is important.

Substantial research has been dedicated to the effects of myocardial ischemia and reperfusion during the hours and days following myocardial ischemia [7–10]. However, the initial pathophysiological effects within the hyperacute phase of acute myocardial ischemia leading to CS are largely unexplored and remain inadequately understood. Even in well-controlled pre-clinical animal models, hyperacute hemodynamic changes post myocardial ischemia are under-reported, with evaluations often occurring hours or days after the ischemic event [11–15]. In addition to detrimental effects on cardiac function and systemic hemodynamics, acute myocardial ischemia complicated by CS impairs mitochondrial oxidative phosphorylation resulting in decreased ATP production and increased anaerobic metabolism, acidosis, and formation of reactive oxygen species (ROS), all of which exacerbate cardiac dysfunction [16, 17]. Despite its potentially critical role in later adverse cardiac remodeling, the temporal evolution of the immediate cardiovascular and mitochondrial effects, and the relation to early hemodynamic malfunction, remains to be explored. A novel understanding of the hemodynamic and cardiac mitochondrial changes in the hyperacute phase of ischemic CS may uncover pathophysiological pathways for future therapeutic strategies.

In this original study, we aimed to elucidate the hyperacute hemodynamic and cardiac mitochondrial effects of ischemic CS induced by microembolization of the left main coronary artery (LMCA) in a large animal model. Using pressure–volume (PV) analysis and endomyocardial biopsies we address the knowledge gap by characterizing immediate hemodynamic instability and mitochondrial perturbations following myocardial ischemia, thereby providing novel insights into early injury mechanisms beyond the scope of prior studies.

## Methods

### Animals

In this experimental study, 32 female Danish Landrace pigs were included. The pigs were intubated and immediately placed under invasive positive-pressure ventilation with a positive end-expiratory pressure of 5 cmH_2_O. Anesthesia maintenance entailed a continuous infusion of propofol (3.5 mg/kg/h) and fentanyl (15 µg/kg/h) as previously described [18–21].

This study was conducted using animals that were part of a previously established research protocol in our laboratory at Aarhus University Hospital, ensuring maximum statistical power while adhering to the 3R principles aimed at minimizing animal use in research. The original research focused on the hemodynamic effects of metabolic substrate treatments during cardiogenic shock complicating myocardial ischemia [20, 21]. The protocol for this study was pre-planned and included in the same ethical approval granted by the Danish National Animal Experiment Inspectorate (Permit no: 2023–15–0201–01466, issued on 19/06–2023). The treatment and ethical oversight of the animals strictly followed established welfare protocols and regulatory standards as mandated by both Danish and European legislation. All methodologies and animal handling procedures were in full compliance with the EU Directive 2010/63/EU on the protection of animals used for scientific purposes.

### Pressure–volume Parameters

A PV admittance catheter (*Transsonic, USA)* was inserted through the common carotid artery and positioned within the LV using fluoroscopic guidance. The catheter was fixated and left untouched for the entire duration of the study period. Prior to data collection, the admittance catheter was calibrated per the manufacturer’s instructions. Pressure-calibration was done by calibrating the catheter to the zero-level by holding the pressure port just below the surface of saline to avoid unwanted pressure effects from the water column above. Volume data were calibrated for each unique animal using SV acquired from a pulmonary artery (PA) catheter (*Swan Ganz, Edwards Lifesciences, USA*). PV measurements were obtained through a PowerLab 8/35 setup (*ADInstruments, Australia)* and were continuously recorded in LabChart 8 Pro (*AD Instruments, Australia*) for offline analysis. Data was collected during respiration. Mean values through three respiratory cycles were used. The following LV parameters were measured [22]: end-systolic pressure (ESP) and volume (ESV), end-diastolic pressure (EDP) and -volume (EDV), ejection fraction (EF), maximal first derivative of pressure (dP/dt(max)), minimum first derivative of pressure (dP/dt(min)), stroke volume (SV), heart rate (HR), Tau, and cardiac output (CO). Initially, in healthy state, a balloon occlusion of the inferior vena cava was performed to acquire the theoretical ventricular volume when no pressure is generated (V_0_) [23, 24]. V_0_ was kept constant over the study period. This allowed for estimation of end-systolic elastance (Ees = ESP/[ESV-V_0_])) [25] which is the slope of the end-systolic PV relationship (ESPVR). Arterial elastance (Ea) was estimated as the slope of the line intersecting at EDV and ESP. Ventriculo-arterial (VA) coupling was calculated as Ea/Ees. Systemic vascular resistance was calculated under the assumption of a constant central venous pressure (CVP) as (SVR = 80 × (ESP-CVP)/CO).

LV energetic parameters were also obtained. Stroke work (SW), which is the area inside the PV-loop and represents the ventricular energy delivered to the arterial system for maintaining forward blood flow, was calculated by LabChart 8 Pro. Potential energy (PE), which is the area of the PV diagram bounded by the ESPVR, the end-diastolic PV relationship and end-systolic portion of the PV loop, was estimated as ESP × (ESV-V_0_)/2 [26], assuming that EDP is small relative to ESP. PE represents the ventricular energy that is not transduced into external work [27]. PVA represents the total mechanical energy during one cardiac cycle as the sum of SW and PE, which is linearly correlated with myocardial oxygen consumption_._[27, 28]. The PVA/SV ratio was calculated to assess the total mechanical energy expenditure per unit of ejected blood. LV cardiac mechanical efficiency (SW/PVA) was calculated. Finally, the total LV mechanical power expenditure (PVA × HR) was estimated [29].

### Systemic and Pulmonary Blood Pressures

Systemic and pulmonary blood pressures; mean arterial blood pressure (MAP), mean pulmonary arterial blood pressure (mPAP), CVP, and pulmonary arterial wedge pressure (PAWP) was assessed using an intraarterial sheath in the femoral artery, and a PA catheter (Swan Ganz, Edwards Lifesciences, USA) respectively. Coronary perfusion pressure was calculated ([diastolic blood pressure]–PAWP).

### Endomyocardial Mitochondrial Respirometry

A flexible biopsy forceps (JawzTM, Argon Medical Devices, USA) was introduced through the left carotid artery and advanced into the LV under fluoroscopic guidance to retrieve endomyocardial biopsies from the interventricular septum. Endomyocardial biopsies were taken at healthy state and after the 60 min period of no-touch after induction of ischemia. Mitochondrial respiratory capacity was measured using the Oxygraph 2 K (Oroboros Instruments, Innsbruck, Austria). First, the substrate protocol included glutamate and malate (GM) to first measure basal GM leak (i.e., proton leak in the absence of ADP) and then state 3 Complex I respiration following ADP addition. Secondly, ADP and succinate were added to evaluate maximal state 3 respiration through both complexes I and II (OXPHOS). Complex II respiration was calculated by subtracting Complex I respiration from the total OXPHOS rate. Respiratory control ratio (RCR) was calculated for complex I, complex II and complex I + II (Complex I RCR = Complex I respiration/GM leak, Complex II RCR = Complex II respiration/GM leak and Complex I + II RCR = OXPHOS/GM leak and), thereby reflecting the efficiency of coupling between oxygen consumption and ATP production. Subsequently, oligomycin was added to evaluate State 4o leak, which reflects the proton leak across the inner mitochondrial membrane when ATP synthase is inhibited. Finally, rotenone and antimycin A were administered to measure residual non-mitochondrial oxygen consumption (ROX). All parameters were corrected for ROX to eliminate non-specific oxygen utilization. Chambers were hyperoxygenated, and all measurements were done in duplicate.

### Blood Samples

Arterial blood samples were collected from the femoral artery. Arterial plasma levels of high-sensitivity troponin I (hs-TnI) were analyzed in batch using a sandwich immunometric high-sensitivity method (Atellica IM, Siemens Healthineers, Germany). The coronary sinus was catheterized through the right jugular vein using a coronary guide catheter under fluoroscopic guidance. Correct positioning of both catheters was ensured using flushes of contrast. Arterial, mixed venous and coronary sinus blood gasses were analyzed immediately after sampling (ABL Flex 90, Denmark).

### Experimental Protocol

**Fig. 1:**
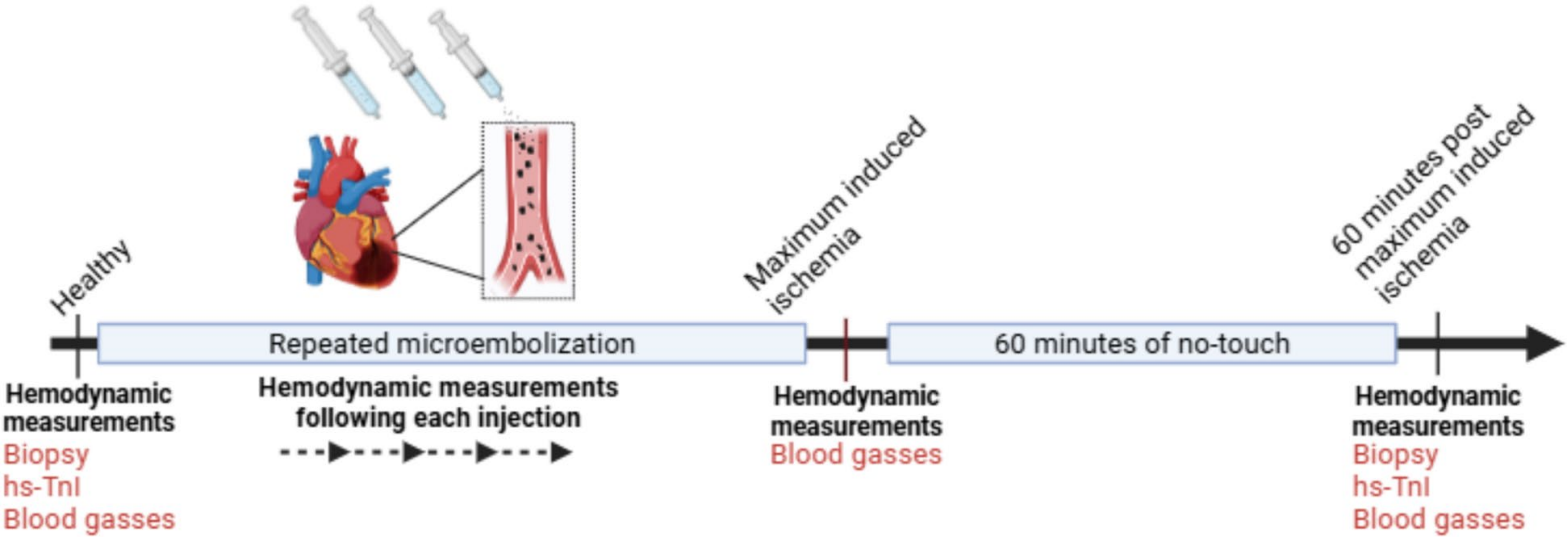
Study design. 30 pigs were included in this study. After instrumentation baseline (healthy state) hemodynamic measurements were conducted and an endomyocardial biopsy was collected. Injections of microspheres were repeatedly administered separated by five-minute intervals until a 30% reduction in cardiac output or mixed venous oxygen saturation was achieved (defined as Maximal induced ischemia). Hemodynamic measurements with a left ventricular pressure–volume catheter were performed following each injection. Upon maximum induced ischemia a 60-min no-touch period was introduced, followed by final hemodynamic measurements and endomyocardial biopsy collection (time point defined as 60-min post maximal induced ischemia)

A large animal model of myocardial ischemia was used [30]. Myocardial ischemia was induced through repeated injections of polyvinyl microspheres (*Contour™, Boston Scientific, USA)* into the LMCA (Fig. 1). The microsphere solution was created by mixing 0.125 g of microspheres with 5 ml of contrast (*Iomeron®, Bracco Imaging, Sweden)* and 5 ml of 0.9% saline (*B.Braun, Denmark).* Microsphere injections were carried out through stepwise injections of 1 ml of microsphere solution. The hemodynamic effects of microsphere-induced myocardial ischemia were assessed after a two-minute stabilization period following each intracoronary injection. Repeated injections were administered at five-minute intervals until either CO measured via thermodilution using a PA catheter or mixed venous oxygen saturation decreased by 30% [19]. This predefined threshold was based on an unpublished pilot study where further injections led to hemodynamic instability requiring vasoactive support. Treatment with vasoactive or inotropic drugs were not allowed in the study protocol.

The pre-defined endpoint was designated as 100% Maximum Induced Ischemia (MII). The point at which half the required microsphere injections to reach MII was administered was defined as 50% MII. To assess hemodynamic response potency in a dose–response-dependent manner, we calculated an R_50_-value as the relative number of emboli needed to induce 50% of the total observed change in a variable following 100% MII. Upon the final injection, animals were left untreated for a 60-min no-touch period before the final hemodynamic measurements were assessed (60-min post-MII). Exclusion criteria was death before the 60-min post-MII. Blood gasses were taken and systemic and pulmonary blood pressures were measured in healthy state, after ischemia induction and following 60 min of no touch.

### Statistical Analysis

The number of animals was predetermined based on the requirements of the original study, ensuring sufficient statistical power to assess the predefined hemodynamic endpoints [20]. Data normality was evaluated using qq-plots and histograms. Temporal changes and the effect of emboli on the measured parameters were assessed using a linear mixed-effects model, with amount of infused emboli as a fixed effect and each animal as a random effect to account for repeated measurements within each animal. Ischemic burden was defined as percentage of emboli required to obtain a CO or mixed venous saturation decrease of 30% as defined in the original protocols [19, 20, 31]. The main table presents the mean ± SD of hemodynamic parameters for normally distributed data, and median and interquartile range (IQR; 25th to 75th percentile) at healthy state, 50% MII, MII and 60-min post-MII. *P*-values for the fixed effect were derived using the Kenward-Roger method, and statistical significance was defined as a two-sided *P*-value < 0.05. Data analysis and graphical representations were performed using R software (*version 4.2.1, RStudio, PBC*) or Prism (*GraphPad Version 8.4.2, GraphPad, USA*).

## Results

Acute myocardial ischemia was induced in 32 animals. Two animals died during ischemia induction due refractory cardiac arrest with ventricular fibrillation. These animals were excluded. Hence, 30 animals were included for analysis in the study. A mean of 4.7 ± 2.1 ml of microsphere solution was required. The mean time of embolization until 100% MII was 23 ± 11 min.

### Hemodynamic Changes in Ischemic Cardiogenic Shock

**Fig. 2:**
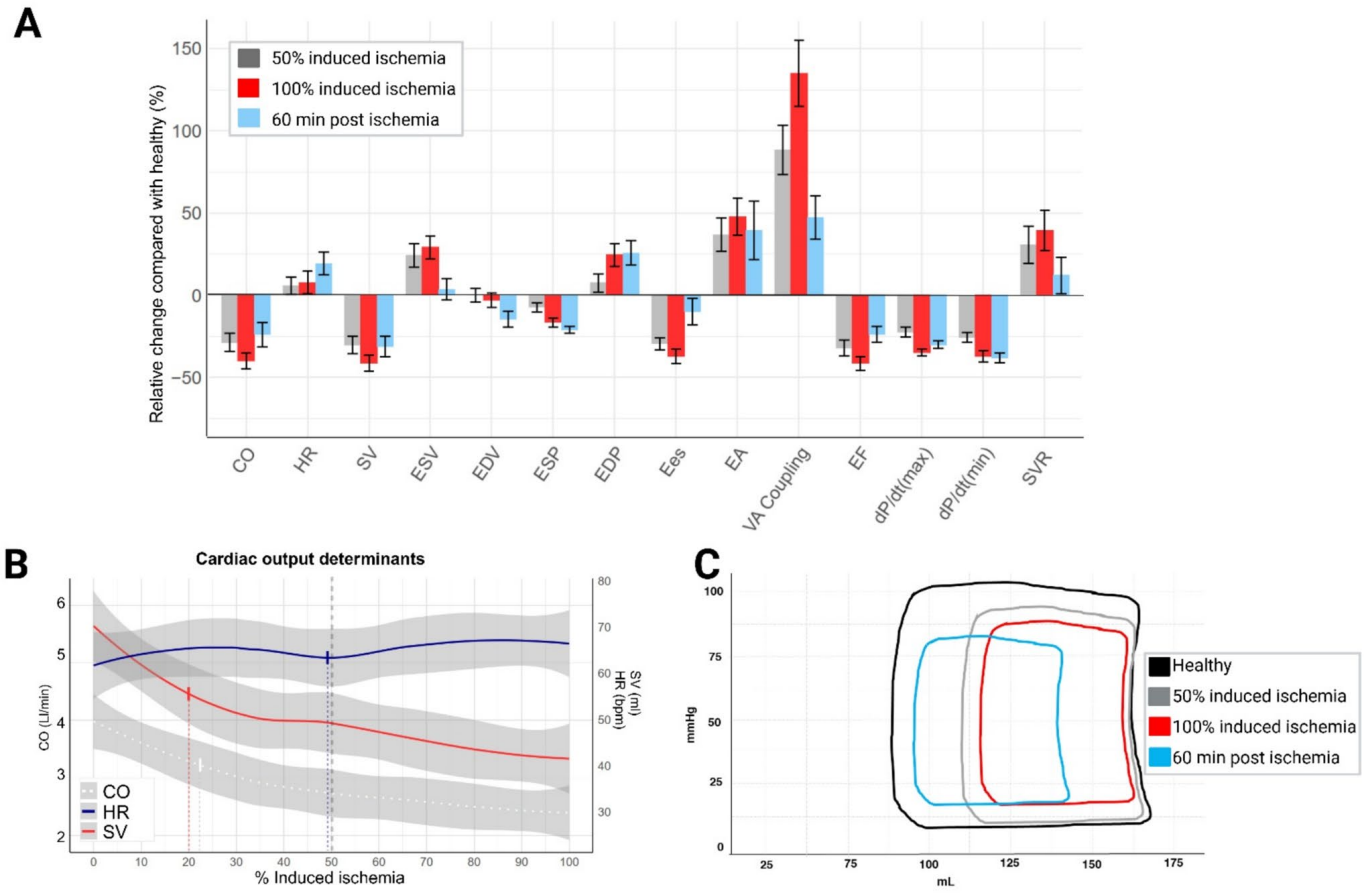
Changes in hemodynamic parameters during embolization. Left ventricular pressure–volume measurements were continuously recorded during the period of embolization. Data was collected during respiration. Mean values through three respiratory cycles were used. **A**: Relative changes in cardiovascular parameters at 50% Maximum induced ischemia(grey), Maximum induced ischemia (red) and 60 min post ischemia (blue) compared to the healthy state. Colored bars represent the mean relative change for each variable, and black error bars indicate the Standard Error of the Mean (SEM). **B**: Relationships between ischemic burden, defined as percentage of induced ischemia and cardiac output (CO) and its determinants heart rate (HR) and stroke volume (SV). All curves were generated using locally weighted scatterplot smoothing (LOESS) to model the trends in the data, with 95% confidence intervals (shaded areas). Vertical lines indicate R_50_ values, indicating the amount of emboli (in %) required to induce half the change observed at 100% Maximal induced ischemia. **C**: Representative left ventricular pressure–volume (PV) loops obtained by the admittance catheter. The figure shows a representative PV loop in healthy state (black), after 50% maximum induced ischemia (grey), 100% maximum induced ischemia(red) and 60 min post ischemia induction (blue). Embolization of the LMCA resulted in a right shift of the PV loops with a decrease in stroke volume and increased filling pressures. CE = cardiac efficiency, CO = cardiac output, Ea = arterial elastance, EDP = end-diastolic pressure, EDV = end-diastolic volume, EF = ejection fraction, Ees = end-systolic elastance, ESP = end-systolic pressure, ESV = end-systolic volume, HR = heart rate, SV = stroke volume, SVR = systemic vascular resistance, VA coupling = ventriculo-arterial coupling (Ea/Ees ratio)

CO showed an immediate decline from 3.9 ± 1.9 L/min in healthy state to 2.8 ± 1.2 L/min after 50% MII and 2.4 ± 1.1 L/min at MII, with a R_50_ value of 22% (Table 1, Fig. 2A-C), indicating rapid deterioration during embolization. Similarly, SV decreased from 62 mL (IQR 47–77 mL) at healthy state to 42 mL (IQR 32–58 mL) after 50% MII and further to 34 mL (IQR 27–45 mL) at MII (R_50_: 20%). HR increased during embolization. 60 min after MII, CO was partly restored to 3.0 ± 1.7 L/min, primarily caused by increased HR.

**Table 1:** Left ventricular hemodynamic alterations during embolization

*N* = *32*	Healthy	50% Maximal induced ischemia	Maximal induced ischemia	60-min post Maximal induced ischemia	R_50_ (%)
**Cardiovascular function (left ventricular parameters)**					
CO (L/min)	3.9 ± 1.9	2.8 ± 1.2*	2.4 ± 1.1‡	3.0 ± 1.7*	22
SV (ml)	62 (47–77)	42 (32–58)†	34 (27–45)‡	37 (29–55)‡	20
HR (bpm)	58 (51–64)	60 (53–75)	60 (50–72)	68 (48–88)*	49
EF (%)	42 ± 14	28 ± 11‡	25 ± 10‡	32 ± 12‡	19
Ees (mmHg/ml)	1.38 (1.06–1.87)	1.02 (0.81–1.26)‡	0.87 (0.69–1.12)‡	1.20 (0.91–1.52)	26
Ea (mmHg/ml)	1.67 (1.28–2.41)	2.31 (1.61–3.18)	2.60 (1.85–3.32)†	2.19 (1.44–3.18)*	26
VA Coupling	1.28 (0.85–1.73)	2.50 (1.60–3.28)‡	2.94 (2.34–4.12)‡	1.70 (1.26–2.12)*	31
dP/dt(max) (mmHg/s)	1602 ± 312	1242 ± 272‡	1045 ± 200‡	1121 ± 235‡	27
dP/dt(min) (mmHg/s)	−2220 ± 424	−1653 ± 391‡	−1391 ± 437‡	−1373 ± 386‡	27
Tau (ms)	43 ± 11	59 ± 13‡	68 ± 17‡	55 ± 14‡	34
SVR, dyne/s/cm^5^	2376 ± 1013	3089 ± 1464*	3312 ± 1641‡	2661 ± 1450	35
**Left ventricular pressures and volumes**					
ESV (mL)	91 ± 36	113 ± 36	118 ± 36*	94 ± 35	24
EDV (mL)	165 ± 39	165 ± 38	160 ± 41	141 ± 48	37
ESP (mmHg)	104 ± 17	96 ± 16†	87 ± 16‡	82 ± 13‡	55
EDP (mmHg)	15 ± 5	16 ± 5	19 ± 6†	19 ± 7†	57
**Left ventricular mechano-energetics**					
SW (mmHg*mL)	5,829 (4,368–7,722)	3,265 (2,673–4,841)‡	2,451 (1,626–3,392)‡	2,528 (2,020–4,130)‡	24
PE (mmHg*mL)	3,705 (3,059–4,566)	4,310 (3,884–5,888)	4,170 (3,439–5,497)	2,565 (1,900–3,199)*	29
PVA (mmHg*mL)	9,818 (7,992–12,063)	8,554 (6,105–10,624)†	6,651 (5,219–8,875)‡	5,079 (4,088–6,319)‡	41
MPE (mmHg*L/min)	619 (387–797)	538 (352–697)	438 (308–585)	366 (292–487)	51
CE (%)	60 ± 11	43 ± 11‡	38 ± 11‡	49 ± 13‡	22
PVA/SV (mmHg)	155 (135–186)	211 (174–277)	183 (162–255)*	138 (108–179)	26

Mean ± standard deviation (SD) or median (25th-75th percentile) values of left ventricular hemodynamic parameters at healthy state, after administration of 50% emboli (50% Maximal induced ischemia) and 100% (Maximal induced ischemia) of emboli necessary to achieve a cardiac output (CO) or mixed venous saturation decrease of 30%, and 60-min post maximum induced ischemia

R_50_ values indicate the amount of emboli (in %) required to induce half the change observed at 100% Maximal induced ischemia and hence points to how quickly alterations during ischemia happens

* indicates *P* < 0.05 compared with Healthy, † indicates *P* < 0.01, ‡ indicates *P* < 0.001

CE = cardiac efficiency, CO = cardiac output, MPE = total LV mechanical power expenditure, Ea = arterial elastance, EDP = end-diastolic pressure, EDV = end-diastolic volume, EF = ejection fraction, Ees = end-systolic elastance, ESP = end-systolic pressure, ESV = end-systolic volume, HR = heart rate, PE = potential energy, PVA = pressure–volume area, SV = stroke volume, SW = stroke work, VA = ventriculo-arterial coupling (Ea/Ees ratio)

**Fig. 3:**
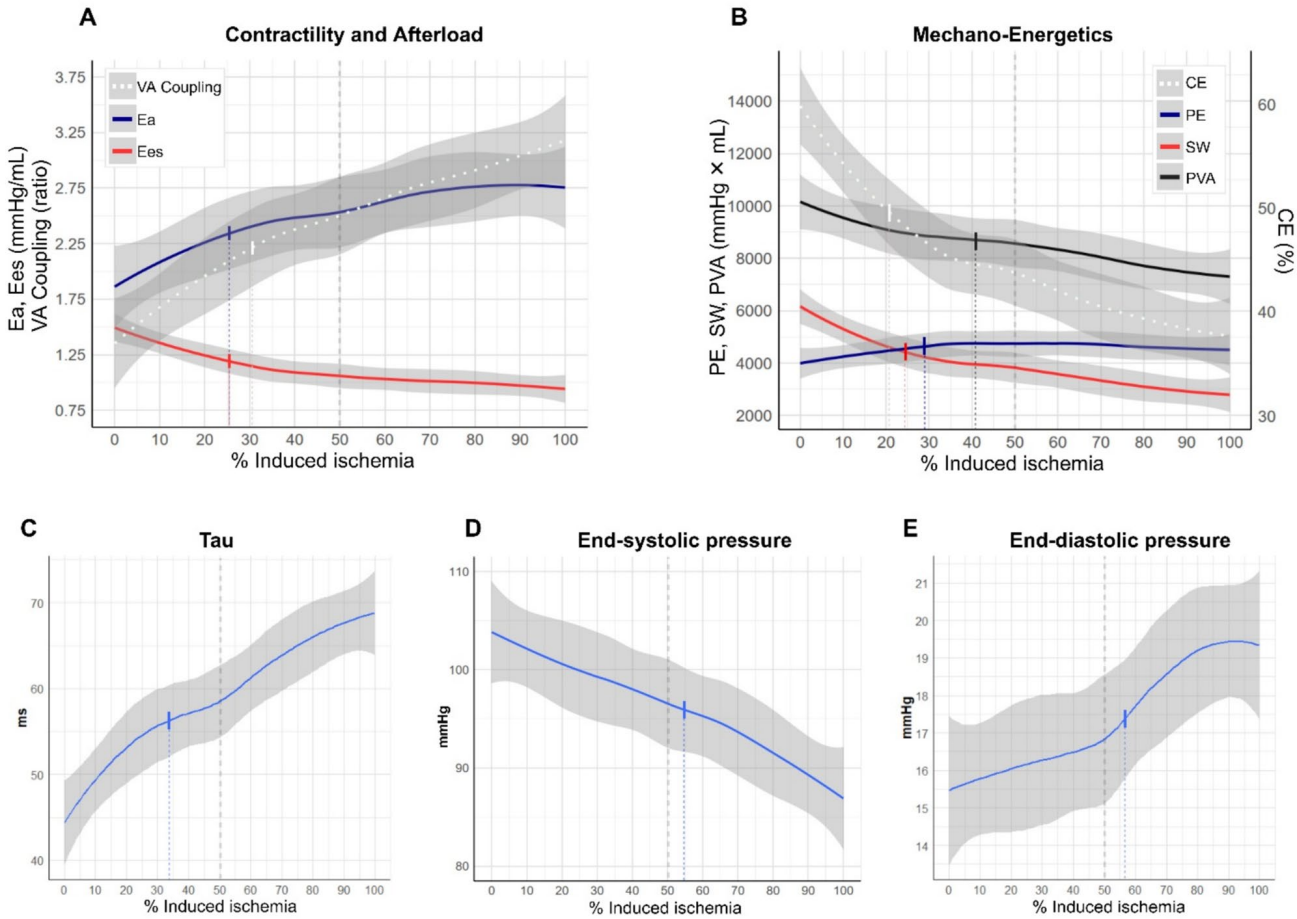
Temporal changes in hemodynamics. Top row: Relationships between ischemic burden and end-systolic elastance (Ees), arterial elastance (Ea), and ventriculo-arterial (VA) coupling (Panel **A**). Relationships between ischemic burden and mechano-energetic parameters pressure volume area (PVA), stroke work (SW) and potential energy (PE) on the left y-axis. On the right y-axis cardiac efficiency (CE) is indicated (Panel **B**). Bottom row: Relationships between ischemic burden and the diastolic function parameter Tau (**C**), End-systolic pressure (**D**), and End-diastolic pressure (**E**). All curves were generated using locally weighted scatterplot smoothing (LOESS) to model the trends in the data, with 95% confidence intervals (shaded areas). Vertical lines indicate R_50_ values, indicating the amount of emboli (in %) required to induce half the change observed at 100% Maximal induced ischemia

Ees decreased from 1.38 mmHg/mL (IQR 1.06–1.87 mmHg/mL) at healthy state to 0.87 mmHg/mL (IQR 0.69–1.12 mmHg/mL) at MII with a low R_50_ value of 26% (Fig. 3A). Similarly, dP/dt(max) decreased. Ea increased from 1.67 mmHg/mL (IQR 1.06–1.87 mmHg/mL) in healthy state to 2.60 mmHg/mL (IQR 1.85–3.32 mmHg/mL) after MII (R_50_: 26%). The decreased Ees and increased Ea resulted in rapid VA decoupling (R_50_: 31%). SVR increased from 2376 ± 1013 dyn/s/cm^5^ in healthy state to 3312 ± 1641 dyn/s/cm^5^ at MII with an R_50_ value of 35%. During 60 min of no touch, Ees was partly restored to 1.20 mmHg/mL (IQR 0.91–1.52 mmHg/mL) and Ea to 2.19 mmHg/mL (IQR 1.44–3.18 mmHg/mL), resulting in incompletely restored VA coupling. SVR was also significantly restored at 60-min post MII, with values not significantly different as in healthy state.

### Left Ventricular Pressure–volume Changes in Ischemic Cardiogenic Shock

The LV surrogate for systemic blood pressure, ESP, decreased from 104 ± 17 mmHg in healthy state, to 87 ± 16 mmHg at MII with a R_50_ value of 55% (Fig. 3D), indicating slower deterioration compared with forward flow parameters. ESP decreased even further 60-min post MII to 82 ± 13 mmHg. EDP increased from 15 ± 5 mmHg in healthy state to 19 ± 6 mmHg at MII, also with a high R_50_ value of 57%, and remained unaltered 60-min post MII (Fig. 3E). The diastolic function parameter Tau increased from 43 ± 11 ms to 68 ± 17 ms at MII, with an R_50_ value of 34% (Fig. 3C). dP/dt(min) also increased. Tau was partly regenerated 60-min post MII. ESV increased during ischemia induction, while EDV remained unchanged. EDV was decreased 60-min post MII while ESV was partly restored.

The external work, SW, decreased during embolization from 5829 mmHg*mL (IQR 4368–7722 mmHg*mL) to 2451 mmHg*mL (IQR 1626–3392 mmHg*mL; Fig. 3B) at MII with a low R_50_ value of 24%. This resulted in a decrease in PVA of 32% and a reduction of total LV mechanical power expenditure of 29% at MII, although with higher R_50_ values. Meanwhile cardiac mechanical efficiency decreased from 60 ± 11% to 38 ± 11%. The PVA/SV ratio, reflecting energy expenditure per unit of stroke volume, was increased during ischemia induction. While cardiac mechanical efficiency was partly restored 60-min post MII, all other energetic parameters declined further.

### Changes in Systemic Hemodynamics and Blood Samples in Ischemic Cardiogenic Shock

MAP decreased during ischemia induction from 89 ± 14 mmHg in healthy state to 71 ± 12 mmHg at MII (Table 2). Arterial, mixed venous, and coronary sinus lactate levels were significantly increased during ischemia induction, indicating an anaerobic shift in systemic and cardiac metabolism. Meanwhile mixed venous oxygen saturation, coronary sinus oxygen saturation, pulse pressure, and coronary perfusion pressure were significantly decreased. The arterio-venous gradient of oxygen across the heart increased during ischemia. The V-A PCO_2_ gap and CS-A PCO_2_ gap both increased during ischemia induction while PAWP increased. No changes were observed in mPAP or CVP.

**Table 2:** Changes in systemic hemodynamics and blood gasses

*N* = *32*	Healthy	Maximal induced ischemia	60-min post maximal induced ischemia
**Arterial blood gasses**			
Oxygen content, mL/dL	12.8 ± 1.2	13.0 ± 1.2	13.3 ± 1.28‡
Oxygen saturation, %	99 (98–100)	99 (98–99)	98 (96–99)
Hemoglobin, mmol/L	5.97 ± 0.59	6.11 ± 0.64	6.31 ± 0.69‡
Glucose, mmol/L	6.5 ± 1.4	7.2 ± 1.7	6.7 ± 1.9
Lactate, mmol/L	1.2 (0.9–1.6)	1.7 (1.4–2.4)‡	2.2 (1.6–2.8)‡
Arterial pH	7.47 ± 0.04	7.46 ± 0.03	7.45 ± 0.04
**Mixed venous blood gasses**			
Oxygen content, mL/dL	7.5 ± 1.5	5.2 ± 1.1‡	5.0 ± 1.0‡
Oxygen saturation, %	57 ± 8	38 ± 7‡	36 ± 6‡
V-A pCO_2_ gap, kPa	1.26 (1.10–1.52)	1.64 (1.48–1.92)‡	1.78 (1.62–1.90)‡
Glucose, mmol/L	6.6 ± 1.5	6.6 ± 1.4	6.7 ± 1.7
Lactate, mmol/L	1.2 (0.9–1.5)	1.6 (1.3–2.4)‡	2.3 (1.7–2.9)‡
**Coronary sinus blood gasses**			
Oxygen content, mL/dL	5.7 ± 2.2	4.0 ± 1.1‡	3.4 ± 1.3‡
Oxygen saturation, %	44.9 ± 18.8	29.7 ± 9.1‡	24.4 ± 9.5‡
CS-A pCO_2_ gap, kPa	1.02 ± 0.54	1.93 ± 0.85‡	1.94 ± 0.61‡
Glucose, mmol/L	6.1 ± 1.4	6.2 ± 1.8	6.0 ± 1.7
Lactate, mmol/L	1.1 (0.7–1.2)	2.3 (1.7–3.2)‡	2.5 (2.0–3.0)‡
**Arterio-venous gradients across the heart**			
Oxygen content gradient, mL/dL	7.5 ± 2.8	9.5 ± 1.4‡	10.3 ± 1.7‡
Glucose gradient, mmol/L	0.4 (0.0–1.0)	0.7 (0.4–1.7)	0.7 (0.3–1.1)
Lactate gradient, mmol/L	0.2 (0.0–0.4)	0.0 (−0.6–0.2)*	−0.2 (−0.4–0.1)
**Systemic hemodynamics**			
Mean arterial blood pressure, mmHg	89 ± 14	71 ± 12‡	69 ± 11‡
Pulse pressure, mmHg	49 (39–56)	39 (31–43)‡	37 (28–43)‡
Central venous pressure, mmHg	6 ± 3	7 ± 3	7 ± 3
Mean pulmonary arterial blood pressure, mmHg	22 ± 7	22 ± 6	24 ± 6
Pulmonary arterial wedge pressure, mmHg	8 (5–9)	12 (10–14)‡	13 (12–15)‡
Coronary perfusion pressure, mmHg	63 ± 15	45 ± 11‡	43 ± 11‡

Mean ± SD or median (25th-75th percentile) values of arterial, mixed venous, and coronary sinus blood gasses, and systemic hemodynamic parameters at healthy state, at maximal induced ischemia, and after 60-min post maximal induced ischemia

* indicates *P* < 0.05 compared with Healthy, † indicates *P* < 0.01, ‡ indicates *P* < 0.001

CS-A pCO_2_ gap = coronary sinus to arterial difference in partial pressure of carbon dioxide, V-A pCO_2_ gap = mixed venous to arterial difference in partial pressure of carbon dioxide

Lactate levels remained higher 60-min post MII while mixed venous and coronary sinus oxygen saturations remained reduced. MAP and coronary perfusion pressure also remained reduced. Hs-TnI was significantly increased after the induction of ischemia (Table 3).

**Table 3:** Changes in mitochondrial function and cardiac troponin I

*n* = 12	Healthy	60-min post maximal induced ischemia	*P*-value
**Mitochondrial active stimulated respiration**			
Complex I respiration (pmol/s/kg)	85 ± 21	77 ± 23	0.048
Complex II respiration (pmol/s/kg)	77 ± 22	59 ± 26	0.080
OXPHOS (pmol/s/kg)	162 ± 37	135 ± 43	0.026
**Mitochondrial respiratory control rates**			
Complex I RCR	14 ± 3	8 ± 3	0.008
Complex II RCR	17 ± 10	8 ± 4	0.012
Complex I + II RCR	25 ± 6	15 ± 6	0.013
**Basal respiration**			
GM leak (pmol/s/kg)	20 ± 4	24 ± 4	0.030
State 4o leak (pmol/s/kg)	46 ± 20	43 ± 20	0.700
ROX	15 ± 3	14 ± 4	0.619
**Oxidative phosphorylation efficiency**			
Complex I, %	44 ± 17	37 ± 22	0.329
Complex II, %	36 ± 14	32 ± 11	0.553
Complex I + II, %	70 ± 8	68 ± 8	0.250
**Arterial biomarker**			
Serum hs-TNI (ng/L)	504 (180–937)	1860 (1395–3135)	0.002

Mean ± SD or median (25th-75th percentile) values of mitochondrial function parameters in before embolization (0% ischemia) and 60 min after full ischemia. The difference between means of mitochondrial data were evaluated using a paired t-test. The difference in hs-TNI was evaluated using a non-parametric Wilcoxon signed rank test

hs-TNI = high sensitivity cardiac troponin I, Leak GM = leak with addition of glutamate and malate, OXPHOS = total oxidative phosphorylation from complex I and II, RCR = respiratory control rate, ROX = residual non-mitochondrial oxygen consumption, State 4o leak = leak across inner mitochondrial membrane after addition of oligomycin with inhibition of ATP synthase

### Changes in Mitochondrial Function in Ischemic Cardiogenic Shock

**Fig. 4:**
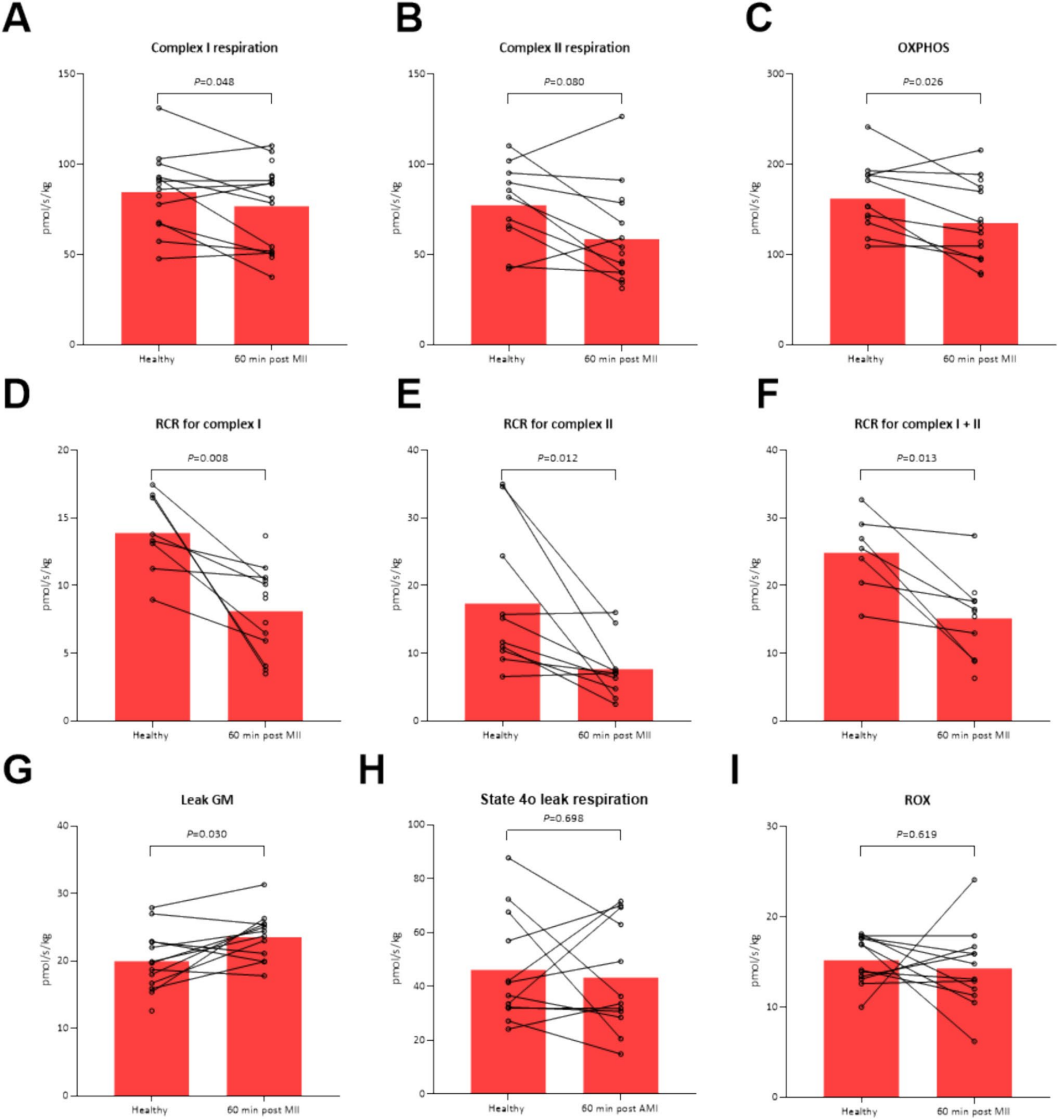
Mitochondrial function in healthy state and after AMI. Panel **A**-**C** shows effects of ischemia on mitochondrial respiration specific to complex I, complex II and complex I + II (OXPHOS). Panel **D**-**F** shows the effects of ischemia on respiratory control ratios (RCR) for complex I, complex II and complex I + II. Panel **G**-**I** shows control efficiency, leak GM and ROX. Individual before-after dots are shown for each individual animal. GM = glutamate and malate, ROX = residual non-mitochondrial oxygen consumption

Myocardial mitochondrial respiration specific to complex I, which reflects electron transport chain activity associated with the reducing equivalent NADH, was significantly decreased in CS (Table 3, Fig. 4A). Mitochondrial respiration specific to complex II was not significantly reduced (*P* = 0.080), while OXPHOS capacity, reflecting fully coupled respiration involving complex I and II (including both reducing equivalents NADH and FADH_2_) was significantly decreased (Fig. 4C). RCR, indicating the efficiency of coupling oxygen consumption and ATP production, was decreased for both complex I and II (*P* = 0.008 and *P* = 0.012 respectively; Fig. 4D-F)). The GM leak, representing substrate-driven, ADP-limited proton leak across the mitochondrial membrane, was significantly increased following ischemia (*P* = 0.030) (Fig. 4G). There was no difference in State 4o leak respiration (*P* = 0.698), representing non-ATP-generating proton leak across the mitochondrial membrane, or ROX (*P* = 0.619), which represents the residual, non-mitochondrial, oxygen consumption after respiratory chain inhibition (Fig. 4H-I).

### Summary

Contractile parameters and forward flow (CO, SV, Ees) responded first to myocardial ischemia, whereas arterial pressure initially remained largely unchanged due to increased vascular resistance. Shortly thereafter, lactate concentrations and veno-arterial pCO₂ gaps rose, evidencing peripheral hypoperfusion and a rapid shift to anaerobic metabolism. Mitochondrial respiration and coupling efficiency declined last, confirming a sequential cascade of early mechanical failure, intermediate metabolic stress, and subsequent mitochondrial dysfunction.

## Discussion

The present study provides a comprehensive investigation of hemodynamics and mitochondrial effects in the hyperacute phase of acute myocardial ischemia leading to CS. This phase was characterized by a rapid decline in systolic and diastolic function and a rapid increase in afterload, resulting in VA decoupling, cardiac energy inefficiency, and decreased mitochondrial function.

Previous preclinical and clinical studies of acute myocardial ischemic injury have mostly assessed injury at later stages in the hours to days following ischemia onset and reperfusion [7–10]. Hence, these studies emphasize fully developed outcomes such as infarct size, cell death, and post-ischemic contractile dysfunction. In contrast, by capturing the hyperacute phase of reperfusion within the very first minutes following onset of ischemia, our study revealed immediate hemodynamic disturbances and mitochondrial dysfunction not detectable in prior studies. Notably, whereas infarct expansion and cardiomyocyte death progress over the initial hours after reperfusion [32], we observed a rapid decline in myocardial contractility accompanied by marked alterations in mitochondrial function occurring almost instantaneously upon ischemic onset. By delineating this insufficiently characterized phase of early injury, our work advances the understanding and knowledge of cardiac pathophysiological changes of myocardial ischemic injury during very early CS. These findings point to the importance of early intervention and treatment strategies.

### Cardiac Dysfunction and Cardiometabolic Impairment Following Ischemic Cardiogenic Shock

We observed rapid systolic dysfunction following the onset of myocardial ischemia as displayed by early reductions in both SV, Ees, EF, and dP/dt(max), all with low R_50_ values. These findings clearly demonstrate a rapid onset of LV contractile dysfunction and compromised forward flow following an ischemic event leading to CS. This substantial contractile deficiency reflects ischemic injury to the myocardium, which becomes deprived of adequate myocellular oxygenation. Diastolic function was also significantly affected. The consequent systemic hypoperfusion activates compensatory sympathoadrenal pathways to cause vasoconstriction, resulting in elevated afterload, as demonstrated by a rapid increase in Ea and SVR [33, 34]. Notably, changes in SVR and Ea preceded blood pressure (ESP) reduction, indicating early compensatory vasoconstriction to maintain end-organ perfusion pressure. However, this also accelerated VA decoupling (increased Ea/Ees ratio) and raised relative myocardial energy demands (increased PVA/SV and decreased mechanical efficiency). Consequently, myocardial oxygen extraction increased, while stroke work decreased, indicating impaired myocardial external efficiency [35]. Thus, oxidative capacity may be exceeded, as evidenced by a shift from net myocardial lactate consumption to production, reflecting anaerobic metabolism.

Hypotension following myocardial ischemia is associated with increased mortality and development of CS and thus, acute therapy and risk stratification are typically guided by blood pressure [36, 37]. However, our findings highlight that flow and volume alterations preceded the development of hypotension. Translating these findings into clinical practice, we therefore emphasize the importance of early evaluation of perfusion parameters, such as stroke volume and LV outflow tract velocity time integral, when evaluating acute myocardial ischemia [36].

At 60-min post MII, we observed an incomplete restoration of cardiac function driven by increased HR. Afterload parameters declined, accompanied by lower systolic blood pressure. This may indicate a shift toward reduced vasoconstriction compared to the hyperacute phase of myocardial ischemia, potentially due to increased activation of the systemic inflammatory response system characteristic of myocardial infarction [38]. At this stage, we also observed an increase in HR which was the main driver to restore CO, while SV did not improve significantly. However, EDP and pulmonary arterial wedge pressure remained elevated, reflecting ongoing cardiac decompensation. Simultaneously, biochemistry perfusion markers SvO_2_ and V-A pCO_2_ gap were not improved 60-min post MII, indicating ongoing peripheral hypoperfusion and microcirculatory imbalance [39–41]. These observations highlight the complexity of CS following acute myocardial ischemia, driven by multiple pathophysiological processes [36].

The dynamic interplay following myocardial ischemia involves rapid cardiac dysfunction, hyperadrenergic compensation [34], and systemic inflammation [38] and underscores the need for individualized therapy based on cardiovascular status. For instance, cardiac unloading benefits conditions with severe cardiac dysfunction and organ hypoperfusion, while treatments that increase myocardial oxygen demand may be harmful [42, 43]. Especially in the very early vulnerable phase of ischemic CS, the heart may be particularly susceptible to further failure, especially if additional stressors are introduced or if compensatory mechanisms falter. For instance, afterload increments through vasopressor treatment may cause harm through increased infarct size, decreased SV, and cardiac mechanical efficiency [44–46]. Conversely, vasoconstrictors may be needed to maintain vital organ perfusion at the expense of decreased perfusion non-vital sections. However, excessive stressors, such as combined inopressors, can further strain the myocardium [47, 48]. Indeed, myocardial mitochondrial respiratory capacities were reduced in the present study, indicating a diminished ability of mitochondria to consume oxygen while synthesizing ATP. In parallel, the drop in RCR reflects a decreased efficiency in ATP generation under maximal energetic demand, suggesting that even the oxygen consumed was converted less efficiently into ATP [49]. Notably, the GM leak across mitochondrial membranes increased following ischemia, potentially pointing to mitochondrial damage and oxidative stress in early phase ischemic CS [16, 17]. The mechanisms behind the mitochondrial damage were beyond the scope of this study. However, it can be speculated that during early ischemia, the abrupt loss of oxygen and substrates halts electron transport and ATP generation, collapsing the mitochondrial membrane potential, suppressing OXPHOS, and precipitating matrix swelling and membrane rupture, events that may directly trigger cell death [50–52]. In addition, mitochondrial ROS surges [53], activation of intrinsic apoptosis pathways [54], Ca^2^⁺-induced permeability-transition pore opening [55], and cardiolipin peroxidation-mediated destabilization of mitochondrial respiratory complexes [56] are contributors that may propagate mitochondrial injury during early ischemia independent of reperfusion. Taken together these observations point to a substantial disruption in mitochondrial function, impairing the energy supply of the heart contributing to post-ischemic contractile dysfunction which may underlie longer term cardiac remodelling [5, 6, 57]. These findings emphasize the potential for novel early mitochondrial targeted therapeutic interventions.

We observed increased Tau (prolonged relaxation time), reduced dP/dt(min), and elevated EDP immediately following myocardial ischemia with low R_50_ values, indicating rapid diastolic dysfunction and acute ventricular stiffness. This resulted in a leftward shift of the PV loop 60 min post MII, compared with 100% MII. While these alterations may be seen as compensatory during CS, protecting against increased wall stress, over time, they predispose the myocardium to a maladaptive remodeling process that may progress to chronic heart failure and dilation of the LV [58–60]. These findings highlight that diastolic dysfunction plays a critical role in the hyperacute phase of ischemia, independent of systolic dysfunction and may serve as a treatment target.

## Limitations

First, the study was conducted using a porcine model, which, although anatomically and physiologically similar to the human heart [61], may not fully replicate the complex pathophysiology of myocardial infarction in humans which may limit the generalizability of the findings to clinical settings. Furthermore, the study was conducted on previously healthy juvenile animals without underlying cardiovascular disease or comorbidities. Also, the model of ischemia induced by microembolization of the LMCA differs from the typical cause of infarction in humans, which is usually due to plaque rupture and thrombosis. While microembolization is a useful method to study ischemia [30], it may not fully capture the dynamic and multifactorial nature of human myocardial infarction, where factors such as reperfusion injury and spontaneous thrombus resolution play significant roles. Microembolization in nature does not allow for a reperfusion phase. Reperfusion can have significant effects on myocardial recovery and the extent of injury, and its exclusion from the experimental protocol limits the applicability of the findings to real-world scenarios where reperfusion therapy is standard. In contrast to reperfused thrombotic occlusion models, our coronary microembolization model induces mitochondrial dysfunction that primarily reflects ischemic injury, devoid of the reperfusion-related oxidative burst and calcium overload known to exacerbate organelle damage [62]. Accordingly, injury to the mitochondria in the present study develops via distinct mechanisms such as progressive substrate deprivation, cumulative metabolic derangement, and mitochondrial uncoupling. Nevertheless, our results demonstrate that prolonged ischemia alone can provoke profound mitochondrial dysfunction, highlighting the pathophysiological importance of these observations even in the complete absence of reperfusion-related stressors. Future studies should aim to validate the findings of this study in clinical settings.

Secondly, the focus on the hyperacute phase of acute myocardial ischemia, while providing novel insights into the immediate hemodynamic changes which remained the sole scope of this study, inherently limits the observation period to the initial minutes post-ischemia. This narrow time frame, which was determined by the original protocols, did not allow for the assessment of longer-term outcomes.

Finally, although the use of permeabilized fibers to assess mitochondrial respiratory capacity is a well-validated, easy method for investigating mitochondrial function with various advantages and high reproducibility, the in-situ preparation presents certain limitations. It results in the absence of cellular context. While high-resolution respirometry provides significant insights into mitochondrial function within a controlled extracellular environment, it does not allow for the evaluation of cellular energetics in intact tissues. Furthermore, samples are subject to damage during isolation. To account for these limitations, all samples were analyzed in duplicate [49].

## Conclusion

This study revealed rapid and significant hemodynamic deterioration during the hyperacute phase of ischemic cardiogenic shock, characterized by systolic and diastolic dysfunction, early mitochondrial damage, and early compensatory mechanisms. These findings highlight the importance of assessing markers of forward flow to identify ongoing hemodynamic deterioration and identify mitochondrial function as a potential treatment target in early ischemic cardiogenic shock.

## Data Availability

The datasets used for analysis in the current study is available from the corresponding author OKH upon reasonable request.
